# Maternal lipids and leptin concentrations are associated with large-for-gestational-age births: a prospective cohort study

**DOI:** 10.1038/s41598-017-00941-y

**Published:** 2017-04-11

**Authors:** Dayana Rodrigues Farias, Lucilla Poston, Ana Beatriz Franco-Sena, Antônio Augusto Moura da Silva, Thatiana Pinto, Lívia Costa de Oliveira, Gilberto Kac

**Affiliations:** 1 Nutritional Epidemiology Observatory, Department of Social and Applied Nutrition, Institute of Nutrition Josué de Castro, Rio de Janeiro Federal University, Rio de Janeiro, Brazil; 2 Division of Women’s Health, King’s College London, St Thomas’ Hospital, London, UK; 3 Public Health Department, Federal University of Maranhão, São Luís, Brazil

## Abstract

The change in maternal lipid, leptin and adiponectin concentrations during pregnancy and infant birth weight (BW) is still poorly characterized. Thus, the aim of the study was to evaluate the association of maternal lipids, leptin and adiponectin throughout pregnancy with large-for-gestational-age (LGA) births and BW z-score. A prospective cohort of 199 mothers was followed during pregnancy in Rio de Janeiro, Brazil. The statistical analyses comprised multiple logistic and linear regression. Women delivered 36 LGA and 11 small-for-gestational-age newborns. HDL-c rate of change throughout pregnancy was negatively associated with BW z-score (β = −1.99; p = 0.003) and the delivery of a LGA newborn (OR = 0.02; p = 0.043). Pregnancy baseline concentration of log leptin was positively associated (OR = 3.92; p = 0.025) with LGA births. LDL-c rate of change throughout pregnancy was positively associated with BW z-score (β = 0.31; p = 0.004). Log triglycerides and log adiponectin were not significantly associated with BW z-score or LGA birth. In conclusion, a higher log leptin pregnancy baseline concentration and a lower HDL-c rate of change during pregnancy were associated with higher odds of having a LGA newborn. These maternal biomarkers are important to foetal growth and could be used in prenatal care as an additional strategy to screen women at risk of inadequate BW.

## Introduction

Birth weight (BW) has been associated with several adverse outcomes, both early and late in life. High BW has been associated with caesarean section, obesity and diabetes later in life^1, 2^. BW is influenced by maternal dietary intake, nutritional status, height, biological markers, gestational age at delivery, smoking habits and socioeconomic and demographic characteristics^3–8^.

Maternal serum cholesterol (HDL-c and LDL-c) is transported across the placenta^9–11^. It is suggested that although the foetal liver synthesizes most foetal cholesterol, maternal cholesterol is crucial in providing cholesterol to the foetus, especially during early pregnancy^9, 10^. Experimental studies indicate that maternal cholesterol affects foetal sterol metabolism and the metabolic functions of extra embryonic foetal tissues, impacting foetal development^12, 13^. Positive associations have been reported between maternal triglycerides (TG) and BW, especially in pregnancies complicated by gestational diabetes, and negative associations have been reported between HDL-c and BW^6, 14–16^. However, some studies have failed to find significant associations. Thus, there is no consensus regarding which lipid fractions are involved and how pregnancy-related changes in lipids may influence BW^6, 14, 17^.

Maternal adiposity is also an important predictor of BW^18^. Maternal blood concentrations of fat-derived hormones, such as leptin and adiponectin, significantly change during pregnancy^18, 19^. However, although an increase in leptin and a decrease in adiponectin concentrations are expected, studies have reported that women experience different rates of change of adipokines during pregnancy depending on pre-pregnancy body mass index (BMI)^20–22^. In this way, the pregnancy change in adipokines could be a potential mechanism linking maternal adiposity to foetal weight gain. Based on these assumptions, associations between maternal leptin and adiponectin concentrations and BW have been investigated, but reports are still contradictory^17, 23^.

Thus, considering the importance of foetal development and subsequent BW for health in early and late life and the lack of consensus regarding the associations between maternal lipids, leptin and adiponectin and BW, the aim of this study was to evaluate the association of maternal lipid, leptin and adiponectin concentrations throughout pregnancy with BW z-score and the prevalence of large-for-gestational-age (LGA) births.

## Results

We evaluated data from 199, 178 and 180 women during the first, second and third gestational trimesters, respectively (9.5% were lost to follow-up). A total of 36 (18.1%) women delivered LGA newborns, whereas 11 were small-for-gestational-age (SGA, 5.5%). The mean gestational age at delivery was 38.8 (SD: 1.7) weeks (data not shown in tables).

Women had a mean age of 26.8 (SD: 5.5) years and an early pregnancy BMI of 25.4 (SD: 4.6) kg/m² at the study baseline. The mean lipid concentrations significantly increased throughout pregnancy: HDL-c by 15.9% (mean increase: 6.9 mg/dL; SD: 8.7); LDL-c by 43.4% (mean increase: 40.1 mg/dL; SD: 26.4); and total cholesterol (TC) by 40.5% (mean increase: 63.0 mg/dL; SD: 32.0). The highest mean percentage increase was observed for TG concentration, 114.8% (mean increase: 79.9 mg/dL; SD: 40.8). Women who delivered LGA newborns presented higher means of early pregnancy BMI (27.0 vs. 25.3 kg/m²; p = 0.042), second trimester glycaemia (82.0 vs. 78.3 mg/dL; p = 0.049) and first trimester leptin concentrations (27.7 vs. 19.6 ng/dL; p = 0.001), as well as lower third trimester adiponectin concentrations (4.6 vs. 6.3 µg/mL; p = 0.045) compared with those who delivered appropriate-for-gestational-age (AGA) newborns (Table 1).

**Table 1:** Pregnancy characteristics according to birth weight categories in a sample of pregnant women and their newborns followed at a public health centre in Rio de Janeiro city, Brazil, 2009–2012.

	Total sample		**SGA** ^1^		**AGA** ^1^		**LGA** ^1^		*p* ^2^	*p* ^3^
	**n**	Mean (SD)	**n**	Mean (SD)	**n**	Mean (SD)	n	Mean (SD)		
Age (years)	199	26.8 (5.5)	11	25.0 (5.9)	152	26.8 (5.6)	36	26.9 (4.9)	0.952	0.290
Education (years of schooling)	199	8.7 (2.9)	11	8.6 (2.0)	152	8.6 (3.0)	36	8.8 (2.6)	0.856	0.987
Early pregnancy BMI (kg/m²)	199	25.4 (4.6)	11	21.8 (2.0)	152	25.3 (4.4)	36	27.0 (5.2)	**0.042**	**0.011**
Pre-pregnancy energy intake (kcal/day)	199	2312 (688)	11	2576 (738)	152	2288 (719)	36	2330 (521)	0.741	0.202
Gestational weight gain (kg)	189	11.8 (4.3)	9	10.6 (3.6)	147	11.6 (4.3)	33	12.5 (4.4)	0.280	0.465
Glycaemia (mg/dL)										
1^st^ T	198	84.3 (9.2)	11	85.5 (7.1)	151	84.5 (9.8)	36	82.8 (7.4)	0.329	0.738
2^nd^ T	171	79.0 (9.6)	8	78.0 (7.9)	131	78.3 (9.6)	32	82.0 (9.7)	**0.049**	0.934
3^rd^ T	174	80.0 (11.8)	9	75.6 (5.4)	136	80.3 (12.5)	29	79.8 (9.2)	0.927	0.262
Mean change (1^st^ to 3^rd^ T)	174	−4.2 (13.7)	9	−11.7 (7.5)	136	−3.8 (14.5)	29	−3.6 (10.8)	0.924	0.112
HDL-cholesterol (mg/dL)										
1^st^ T	199	47.9 (8.2)	11	44.7 (6.5)	152	47.9 (8.3)	36	49.1 (7.6)	0.401	0.224
2^nd^ T	178	57.4 (9.9)	9	57.7 (11.7)	137	57.6 (9.9)	32	56.6 (9.7)	0.617	0.991
3^rd^ T	180	54.8 (9.9)	10	57.0 (10.8)	141	55.0 (9.8)	29	53.3 (10.3)	0.398	0.542
Mean change (1^st^ to 3^rd^ T)	180	6.9 (8.7)	10	11.1 (6.0)	141	7.0 (8.6)	29	4.3 (3.3)	0.215	0.169
LDL-cholesterol (mg/dL)										
1^st^ T	199	96.0 (21.1)	11	85.6 (21.8)	152	96.3 (21.9)	36	97.9 (16.1)	0.690	0.120
2^nd^ T	178	127.0 (28.7)	9	106.6 (27.7)	137	126.3 (28.3)	32	135.9 (27.9)	0.085	**0.046**
3^rd^ T	180	136.7 (33.2)	10	107.2 (31.0)	141	137.6 (33.0)	29	142.3 (30.7)	0.481	**0.005**
Mean change (1^st^ to 3^rd^ T)	180	40.1 (26.4)	10	24.9 (18.6)	141	40.7 (26.2)	29	42.2 (28.5)	0.778	0.080
Total cholesterol (mg/dL)										
1^st^ T	199	159.8 (27.8)	11	144.7 (26.7)	152	160.3 (29.2)	36	162.5 (20.3)	0.675	0.088
2^nd^ T	178	210.8 (35.8)	9	190.6 (35.9)	137	210.2 (35.9)	32	218.8 (33.5)	0.218	0.114
3^rd^ T	180	223.2 (40.8)	10	194.9 (39.8)	141	224.3 (40.8)	29	227.6 (39.0)	0.686	**0.029**
Mean change (1^st^ to 3^rd^ T)	180	63.0 (32.0)	10	53.1 (23.6)	141	63.3 (31.6)	29	64.6 (36.1)	0.848	0.344
Triglycerides (mg/dL)										
1^st^ T	199	79.5 (31.9)	11	71.8 (31.4)	152	80.6 (33.8)	36	77.2 (22.7)	0.569	0.406
2^nd^ T	178	131.6 (48.9)	9	131.3 (58.5)	137	131.6 (50.8)	32	131.6 (38.2)	0.995	0.986
3^rd^ T	180	158.2 (49.4)	10	153.4 (60.5)	141	158.2 (51.3)	29	160.0 (35.9)	0.859	0.777
Mean change (1^st^ to 3^rd^ T)	180	79.9 (40.8)	10	85.2 (48.9)	141	78.0 (41.5)	29	87.8 (34.5)	0.234	0.616
Leptin (ng/dL)										
1^st^ T	199	20.7 (13.7)	11	14.5 (11.5)	152	19.6 (12.7)	36	27.7 (16.2)	**0.001**	0.200
2^nd^ T	178	33.0 (20.2)	9	34.0 (24.4)	137	31.4 (19.6)	32	38.2 (21.3)	0.103	0.746
3^rd^ T	178	32.2 (21.7)	10	36.2 (36.1)	139	31.0 (20.2)	29	36.6 (22.8)	0.184	0.461
Mean change (1^st^ to 3^rd^ T)	178	11.7 (18.0)	10	17.9 (32.8)	139	11.4 (17.0)	29	11.0 (17.1)	0.902	0.301
Adiponectin (µg/mL)										
1^st^ T	199	6.0 (3.9)	11	7.5 (4.3)	152	6.1 (3.4)	36	5.3 (5.4)	0.272	0.177
2^nd^ T	173	7.0 (5.9)	9	5.7 (2.8)	132	7.4 (6.3)	32	6.0 (4.3)	0.255	0.438
3^rd^ T	177	6.1 (4.2)	10	8.8 (7.2)	138	6.3 (4.2)	29	4.6 (2.0)	**0.045**	0.083
Mean change (1^st^ to 3^rd^ T)	177	0.6 (3.6)	10	2.8 (6.6)	138	0.51 (3.6)	29	0.04 (2.0)	0.499	0.075

^1^Birth weight was classified according to the international foetal and newborn growth consortium for the 21^st^ Century (Intergrowth-21^st^) curves. ^2^p-value refers to Student’s t test comparison between AGA and LGA. ^3^p-value refers to Student’s t test for comparison between AGA and SGA.

AGA = adequate for gestational age; BMI = Body mass index; HDL-c = high density lipoprotein; LDL-c = low density lipoprotein; LGA = large for gestational age; SD = standard deviation; SGA = small for gestational age; T = trimester.

Seventy-six (40.4%) women were classified as overweight or obese, 12 (6.0%) smoked during the first trimester, and 112 (56.3%) reported >8 years of schooling. We observed a higher frequency of LGA births among women with early pregnancy overweight or obesity (55.9%) compared to those with normal weight (38.2%) (Table 2).

**Table 2:** Distribution of maternal characteristics according to birth weight categories in a sample of pregnant women and their newborns followed at a public health centre in Rio de Janeiro city, Brazil, 2009–2012.

Variables	Total sample		SGA^1^		AGA^1^		LGA^1^		*p* ^2^	***p*** ^3^
	N	%	n	%	n	%	n	%		
Age (years)										
≤30	141	70.8	9	6.4	105	74.5	27	19.1	0.545	0.508
>30	58	29.2	2	3.4	47	81.0	9	15.5		
Education (years of schooling)										
≤8	87	43.7	5	5.8	68	78.2	14	16.1	0.519	1.000
>8	112	56.3	6	5.4	84	75.0	22	19.6		
Smoking habit										
No	187	94.0	10	5.4	141	75.4	36	19.2	0.093	0.581
Yes	12	6.0	1	8.3	11	91.2	0	0.0		
Alcohol consumption										
No	163	81.9	9	5.5	124	76.1	30	18.4	0.806	1.000
Yes	36	18.1	2	5.6	28	77.8	6	16.7		
Pre-pregnancy LTPA										
No	147	74.6	9	6.1	115	78.2	23	15.7	0.102	1.000
Yes	50	25.4	2	4.0	35	70.0	13	26.0		
Parity (parturitions)										
0	77	38.7	7	9.1	60	77.9	10	13.0	0.137	0.202
≥1	122	61.3	4	3.3	92	75.4	26	21.3		
Pre-pregnancy BMI (kg/m²)										
18.5 - 24.9	112	59.6	8	7.1	89	79.5	15	13.4	**0.042**	0.325
≥25.0	76	40.4	2	2.6	55	72.4	19	25.0		

^1^Birth weight was classified according to the international foetal and newborn growth consortium for the 21^st^ Century (Intergrowth-21^st^) curves. ^2^p-value refers to chi-square or Fisher’s exact test for proportions between AGA and LGA; ^3^p-value refers to chi-square or Fisher’s exact test for proportions between AGA and SGA.

AGA = adequate for gestational age; BMI = body mass index; LGA = large for gestational age; LTPA = leisure time physical activity; SD = standard deviation; SGA = small for gestational age.

**Figure 1:**
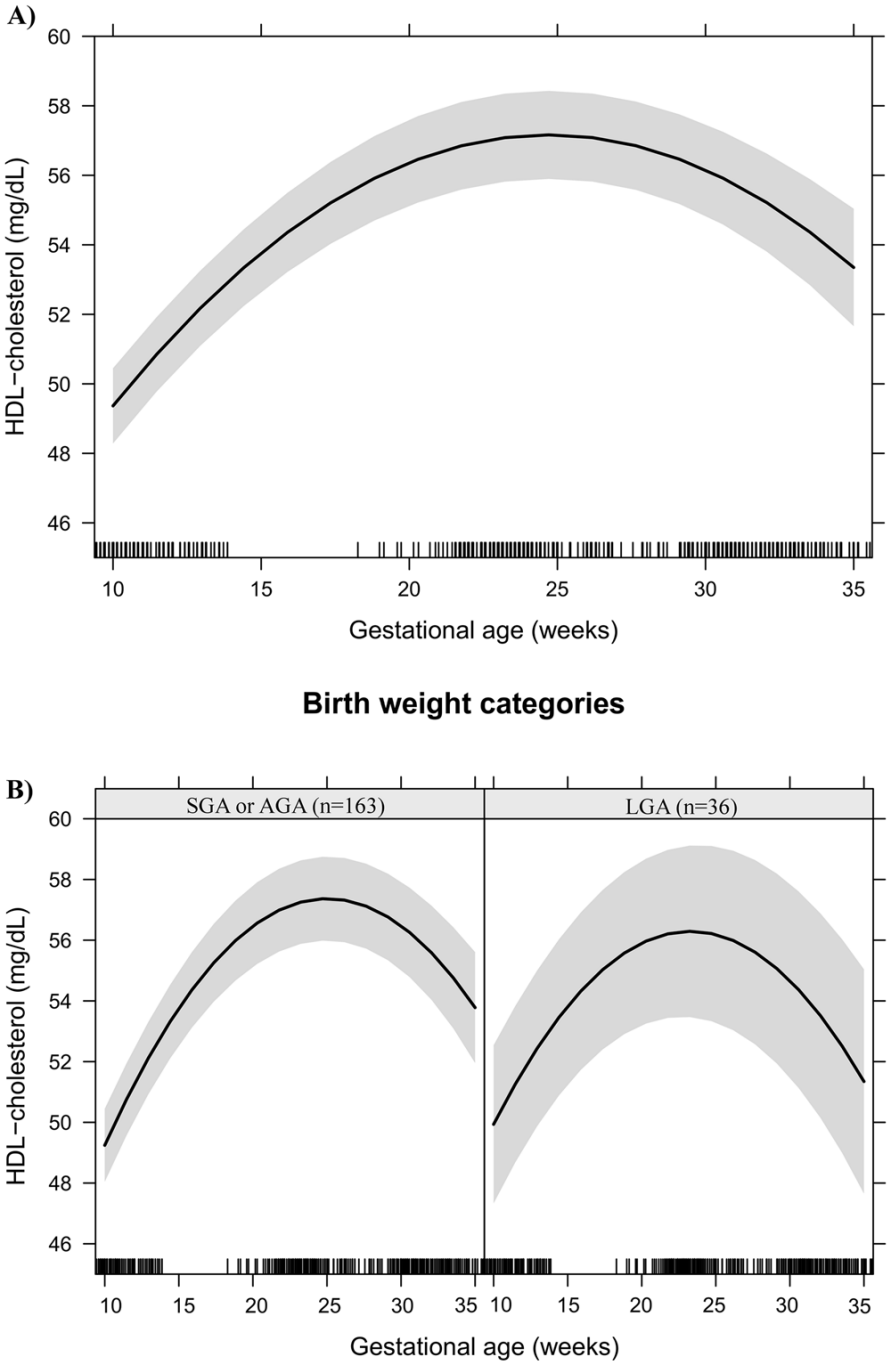
HDL-cholesterol changes during pregnancy in a sample of 199 women and their newborns followed at a public health centre in Rio de Janeiro city, Brazil, 2009–2012. (**A**) Change per gestational week. (**B**) Change per gestational week according to birth weight categories. Note: The models were adjusted for age, education, smoking habit, pre-pregnancy leisure time physical activity, pre-pregnancy energy intake, glycaemia, parity, early pregnancy BMI, gestational weight gain and quadratic gestational age. Data are presented as linear mixed effect coefficient (β) and 95% CI. p-value refers to the maximum likelihood estimator. (**A**) Linear gestational age: β = 1.80 (1.52; 2.08), p < 0.001; Quadratic gestational age: β = −0.04 (−0.04; −0.03), p < 0.001. (**B**) **SGA or AGA** - Linear gestational age: β = 1.76 (1.44; 2.08), p < 0.001; quadratic gestational age; β = −0.04 (−0.04; −0.03), p < 0.001. **LGA** - Linear gestational age: β = 2.04 (1.42; 2.65), p < 0.001; gestational age; β = −0.04 (−0.06; −0.03), p < 0.001.

**Figure 2:**
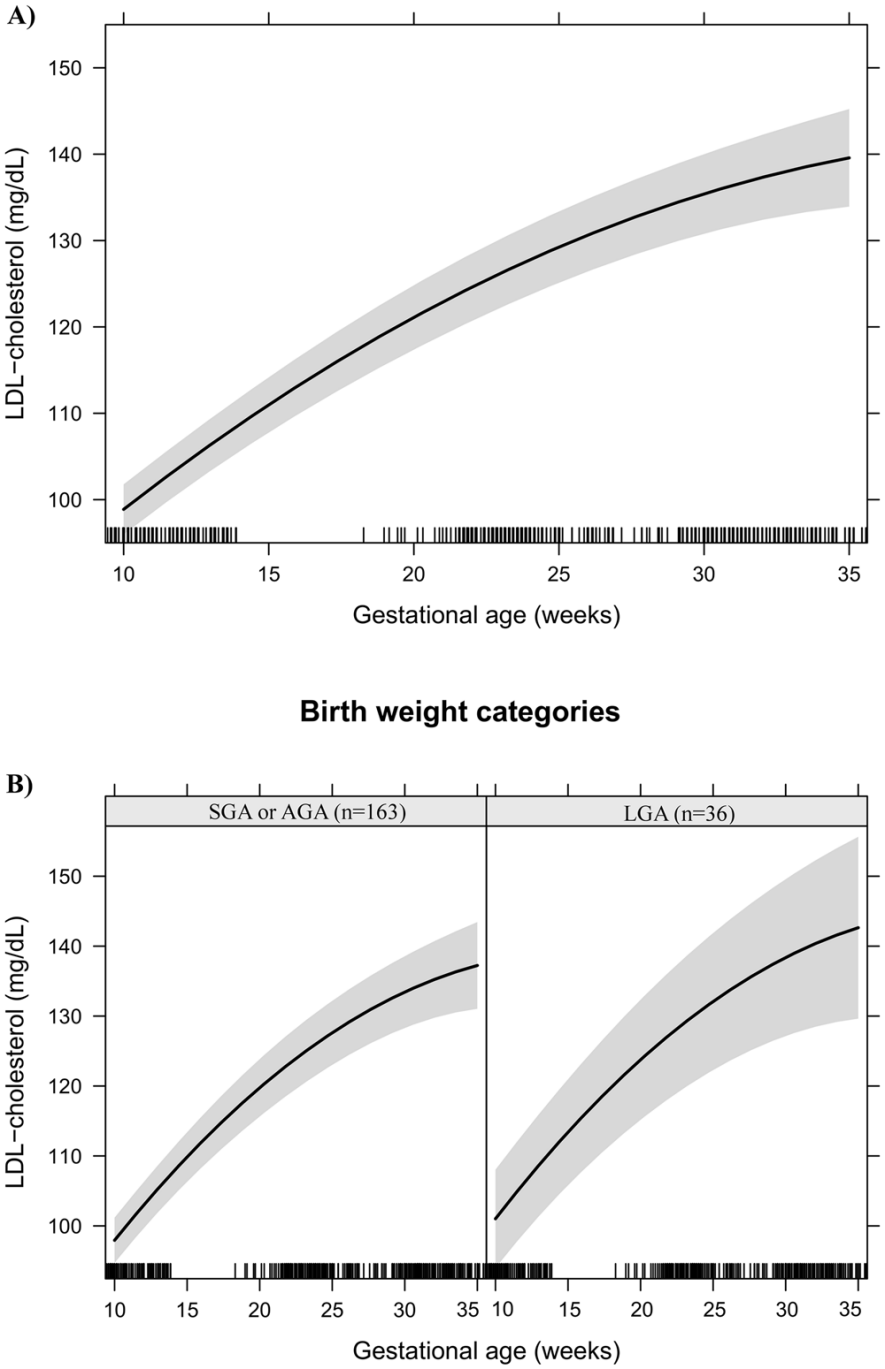
LDL-cholesterol changes during pregnancy in a sample of 199 women and their newborns followed at a public health centre in Rio de Janeiro city, Brazil, 2009–2012. (**A**) Change per gestational week. (**B**) Change per gestational week according to birth weight categories. Note: The models were adjusted for age, education, smoking habit, pre-pregnancy leisure time physical activity, pre-pregnancy energy intake, glycaemia, parity, early pregnancy BMI, gestational weight gain and quadratic gestational age. Data are presented as linear mixed effect coefficient (β) and 95% CI. p-value refers to the maximum likelihood estimator. (**A**) Linear gestational age: β = 3.46 (2.77; 4.14), p < 0.001; Quadratic gestational age: β = −0.042 (−0.06; −0.02), p < 0.001. (**B**) **SGA or AGA** - Linear gestational age: β = 3.11 (2.38; 3.83), p < 0.001; quadratic gestational age; β = −0.03 (−0.05; −0.02), p < 0.001. **LGA** - Linear gestational age: β = 4.956 (3.30; 6.62), p < 0.001; gestational age; β = −0.08 (−0.12; −0.04), p < 0.001.

**Figure 3:**
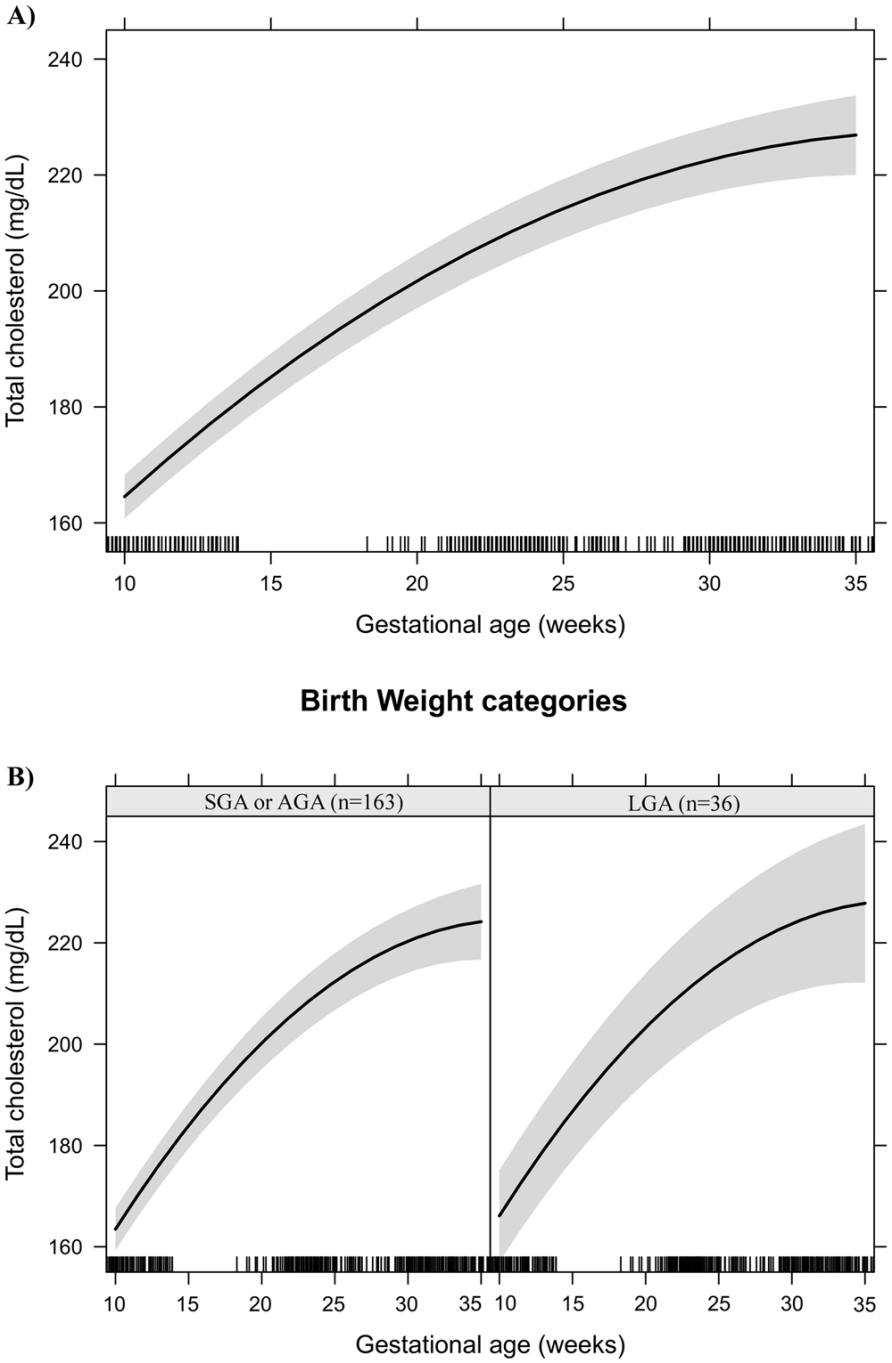
Total cholesterol changes during pregnancy in a sample of 199 women and their newborns followed at a public health centre in Rio de Janeiro city, Brazil, 2009–2012. (**A**) Change per gestational week. (**B**) Change per gestational week according to birth weight categories. Note: The models were adjusted for age, education, smoking habit, pre-pregnancy leisure time physical activity, pre-pregnancy energy intake, glycaemia, parity, early pregnancy BMI, gestational weight gain and quadratic gestational age. Data are presented as linear mixed effect coefficient (β) and 95% CI. p-value refers to the maximum likelihood estimator.(**A**) Linear gestational age: β = 6.21 (5.36; 7.05), p < 0.001; Quadratic gestational age: β = −0.085 (−0.10; −0.07), p < 0.001. (**B**) **SGA or AGA** - Linear gestational age: β = 5.88 (4.98; 6.78), p < 0.001; quadratic gestational age; β = −0.08 (−0.10; −0.06), p < 0.001. **LGA** - Linear gestational age: β = 7.67 (5.50; 9.83), p < 0.001; gestational age; β = −0.12 (−0.17; −0.07), p < 0.001.

**Figure 4:**
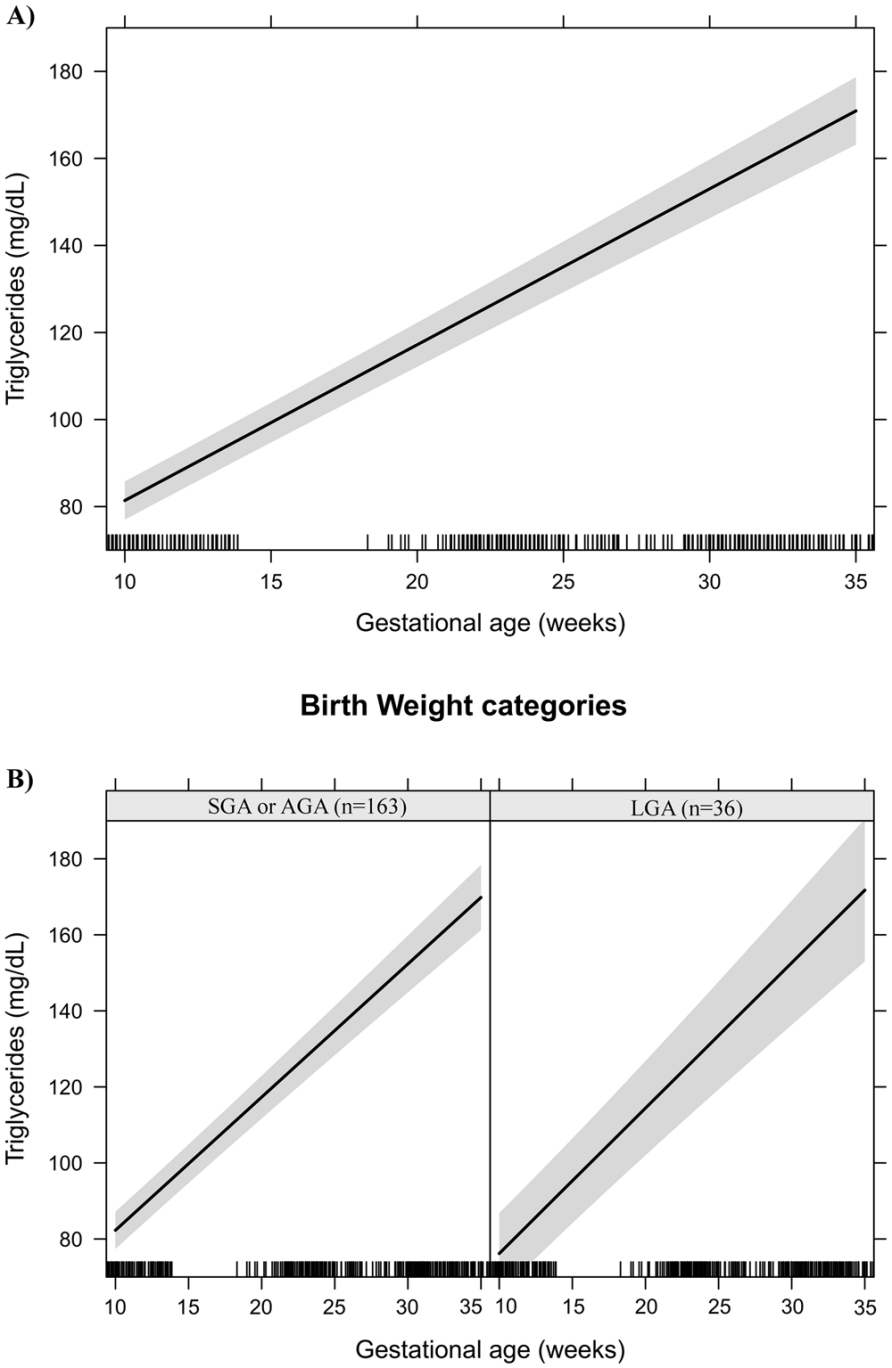
Triglycerides changes during pregnancy in a sample of 199 women and their newborns followed at a public health centre in Rio de Janeiro city, Brazil, 2009–2012. (**A**) Change per gestational week. (**B**) Change per gestational week according to birth weight categories. Note: The models were adjusted for age, education, smoking habit, pre-pregnancy leisure time physical activity, pre-pregnancy energy intake, glycaemia, parity, early pregnancy BMI, gestational weight gain and quadratic gestational age. Data are presented as linear mixed effect coefficient (β) and 95% CI. p-value refers to the maximum likelihood estimator. (**A**) Linear gestational age: β = 3.52 (3.26; 3.78), p < 0.001. (**B) SGA or AGA** - Linear gestational age: β = 3.45 (3.16; 3.74), p < 0.001. **LGA** - Linear gestational age: β = 3.79 (3.23; 4.35), p < 0.001.

**Figure 5:**
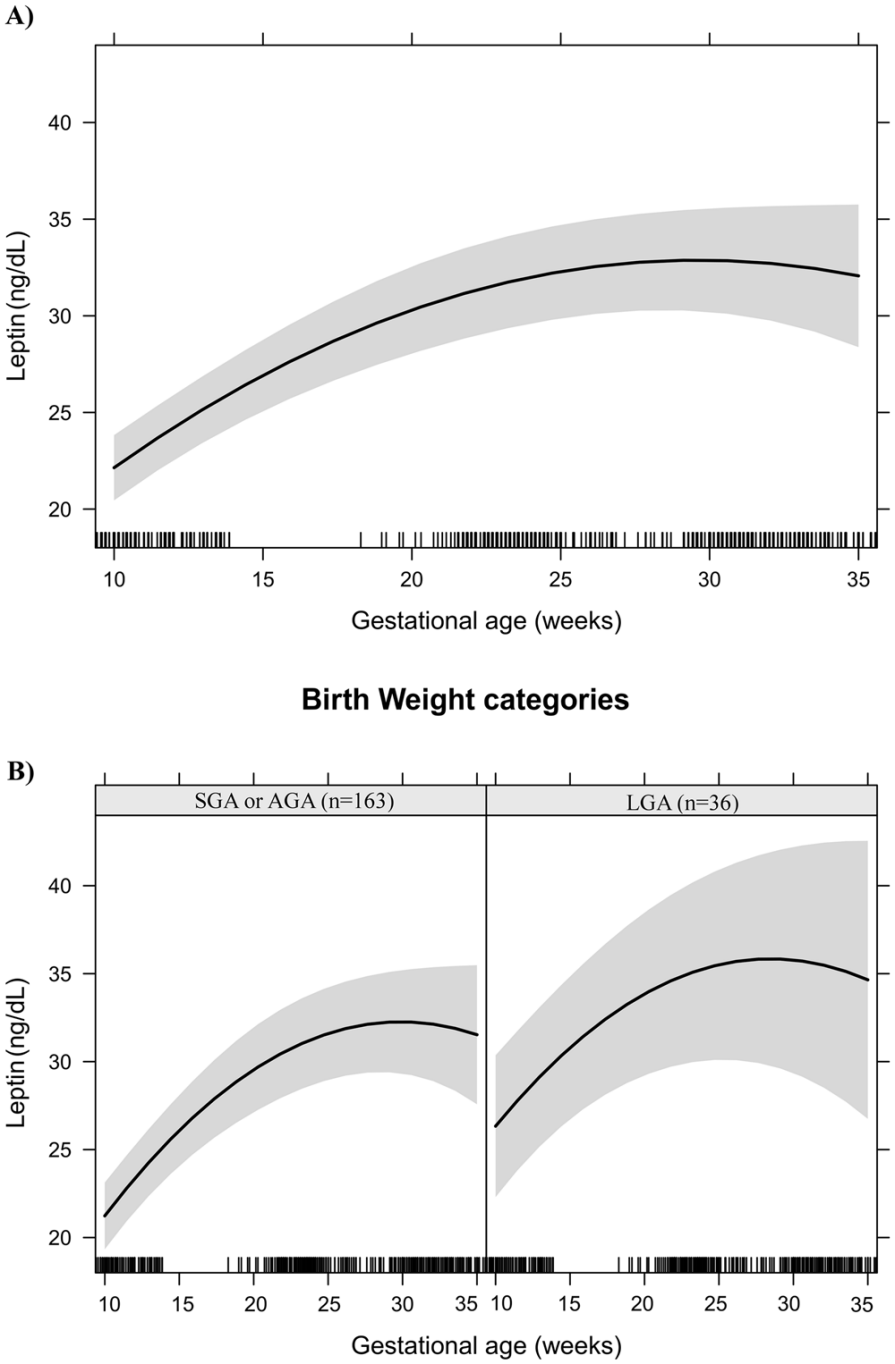
Leptin changes during pregnancy in a sample of 199 women and their newborns followed at a public health centre in Rio de Janeiro city, Brazil, 2009–2012. (**A**) Change per gestational week. (**B**) Change per gestational week according to birth weight categories. Note: The models were adjusted for age, education, smoking habit, pre-pregnancy leisure time physical activity, pre-pregnancy energy intake, glycaemia, parity, early pregnancy BMI, gestational weight gain and quadratic gestational age. Data are presented as linear mixed effect coefficient (β) and 95% CI. p-value refers to the maximum likelihood estimator. (**A**) Linear gestational age: β = 1.60 (0.98; 2.22), p < 0.001; Quadratic gestational age: β = −0.027 (−0.04; −0.01), p = 0.001. (**B**) **SGA or AGA** - Linear gestational age: β = 1.74 (1.07; 2.42), p < 0.001; quadratic gestational age; β = −0.029 (−0.04; −0.01), p < 0.001. **LGA** - Linear gestational age: β = 1.33 (−0.14; 2.81), p = 0.077; gestational age; β = −0.02 (−0.06; −0.01), p = 0.233.

**Figure 6:**
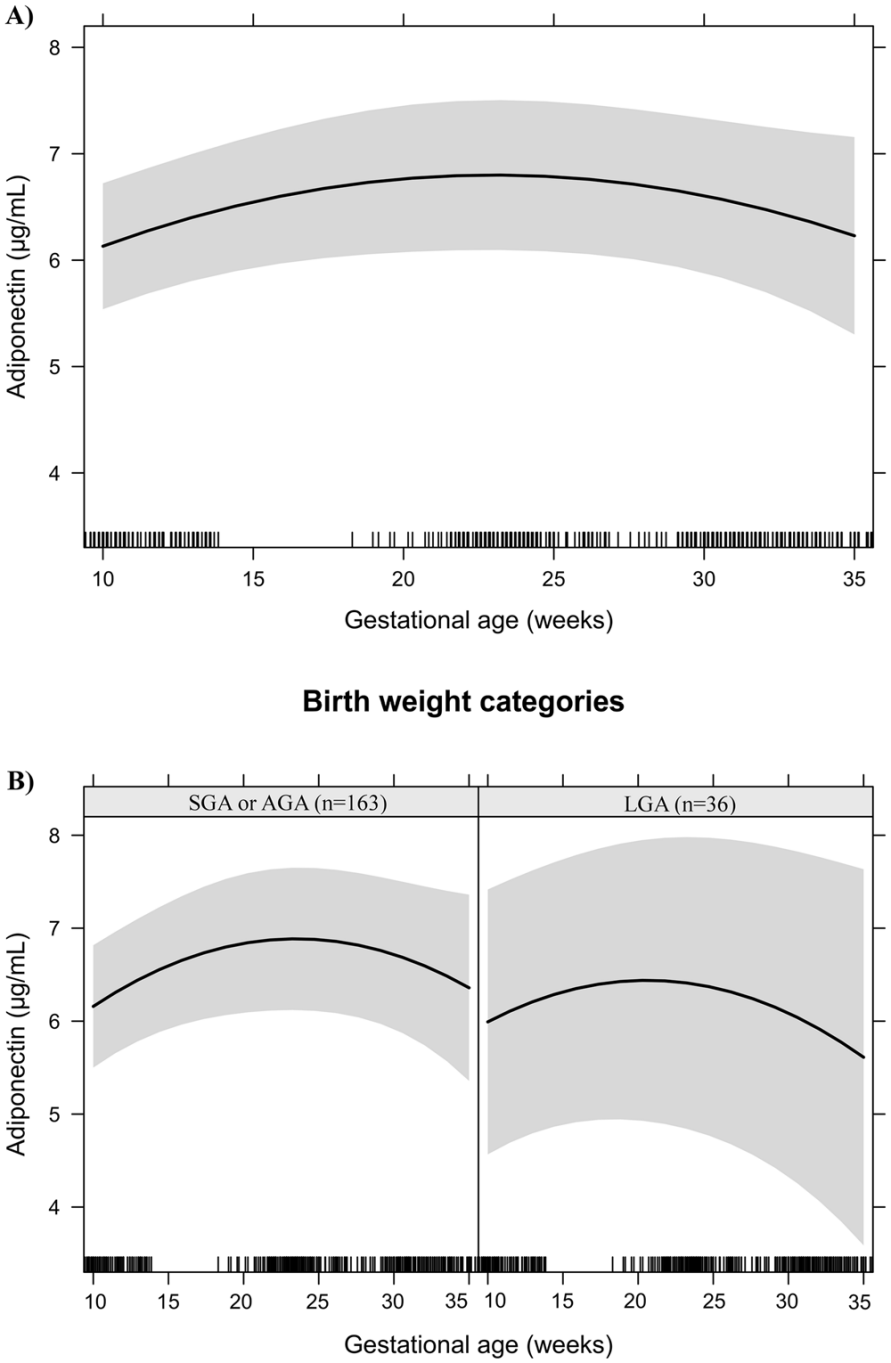
Adiponectin changes during pregnancy in a sample of 199 women and their newborns followed at a public health centre in Rio de Janeiro city, Brazil, 2009–2012. (**A**) Change per gestational week. (**B**) Change per gestational week according to birth weight categories. Note: The models were adjusted for age, education, smoking habit, pre-pregnancy leisure time physical activity, pre-pregnancy energy intake, glycaemia, parity, early pregnancy BMI, gestational weight gain and quadratic gestational age. Data are presented as linear mixed effect coefficient (β) and 95% CI. p-value refers to the maximum likelihood estimator. (**A**) Linear gestational age: β = 0.19 (0.03; 0.34), p = 0.018; Quadratic gestational age: β = −0.004 (−0.01; −0.0003), p = 0.033. (**B**) **SGA or AGA** - Linear gestational age: β = 0.21 (0.03; 0.39), p = 0.021; quadratic gestational age; β = −0.004 (−0.01; −0.0002), p = 0.042. **LGA** - Linear gestational age: β = 0.13 (−0.02; 0.29), p = 0.096; gestational age; β = −0.004 (−0.01; 0.0003), p = 0.031.

Figures 1–6 present longitudinal changes in the maternal biomarkers stratified according to BW category. The analyses revealed that the HDL-c rate of change per gestational week was significantly lower in the LGA group compared to the AGA and SGA groups (Fig. 1). We did not observe significant differences in longitudinal changes of LDL-c (Fig. 2), TC (Fig. 3), TG (Fig. 4), leptin (Fig. 5) or adiponectin (Fig. 6) between BW groups. However, women who delivered LGA newborns presented higher early pregnancy leptin concentrations compared with those who delivered AGA or SGA newborns (Fig. 5).

Mean BW z-scores tended to be higher in women who practiced leisure time physical activity (LTPA) before pregnancy than in those who did not (0.52 vs. 0.25; p = 0.119), with one or more parturitions than in nulliparous (0.42 vs. 0.14; p = 0.072), and in women with overweight/obesity than in those with a normal weight at early pregnancy (0.48 vs. 0.22; p = 0.103) (Table 3).

**Table 3:** Mean birth weight z-score according to maternal characteristics in a sample of pregnant women and their newborns followed at a public health centre in Rio de Janeiro city, Brazil, 2009–2012.

Variables	N	Birth weight z-score^1^	
		Mean (SD)	*p* ^2^
Age (years)			
≤30	141	0.28 (1.09)	0.470
>30	58	0.40 (1.01)	
Education (years of schooling)			
≤8	87	0.30 (1.03)	0.893
>8	112	0.32 (1.09)	
Smoking habit			
No	187	0.33 (1.07)	0.210
Yes	12	−0.06 (0.98)	
Alcohol consumption			
No	163	0.32 (1.05)	0.835
Yes	36	0.28 (1.13)	
Pre-pregnancy LTPA			
No	147	0.25 (1.00)	0.119
Yes	50	0.52 (1.22)	
Parity (parturitions)			
0	77	0.14 (1.08)	0.072
≥1	122	0.42 (1.04)	
Pre-pregnancy BMI (kg/m²)			
18.5–24.9	112	0.22 (1.07)	0.103
≥25.0	76	0.48 (1.05)	

^1^Birth weight z-score was classified according to the international foetal and newborn growth consortium for the 21^st^ Century (Intergrowth-21^st^) curves. ^2^p-value refers to unpaired Student’s *t* test.

**Abbreviations:** BMI = body mass index; LGA = large for gestational age; LTPA = leisure time physical activity; SD = standard deviation.

HDL-c rate of change during pregnancy was negatively associated with LGA birth (slope OR = 0.01; 95%CI: 0.0002 to 0.29; p = 0.009), while pregnancy baseline concentrations of log leptin were positively associated with LGA birth (intercept OR = 3.88; 95%CI: 1.49 to 10.09; p = 0.005) (Table 4). The association between HDL-c (rate of change/slope OR = 0.02; 95%CI: 0.0003 to 0.88; p = 0.043) and log leptin (pregnancy baseline concentrations/intercept OR = 3.92; 95%CI: 1.18 to 12.95; p = 0.025) with LGA births persisted even in the fully adjusted model (Table 4 **, model 3**).

**Table 4:** Logistic regression between maternal lipids, log leptin and log adiponectin time trends during pregnancy and large-for-gestational-age (LGA)^1^ in a sample of women and their newborns followed at a public health centre in Rio de Janeiro city, Brazil, 2009–2012.

	LGA^1^					
	Model 1 (n = 199)		Model 2 (n = 186)		Model 3 (n = 186)	
	OR^2^ (95% CI)	p^3^	OR^2^ (95% CI)	p^3^	OR^2^ (95% CI)	p^3^
HDL-cholesterol						
Intercept	1.11 (1.00 to 1.24)	0.050	1.09 (0.96 to 1.23)	0.170	1.10 (0.97 to 1.24)	0.135
Slope	0.01 (0.0002 to 0.29)	**0.009**	0.02 (0.0004 to 1.09)	**0.055**	0.02 (0.0003 to 0.88)	**0.043**
LDL-cholesterol						
Intercept	0.99 (0.95 to 1.03)	0.553	1.00 (0.96 to 1.04)	0.898	0.99 (0.95 to 1.03)	0.751
Slope	1.69 (0.95 to 2.99)	0.073	1.46 (0.77 to 2.74)	0.245	1.52 (0.80 to 2.88)	0.203
Log triglycerides						
Intercept	0.96 (0.19 to 4.83)	0.965	1.30 (0.23 to 7.42)	0.772	1.09 (0.18 to 6.51)	0.922
Slope	4.5e + 34 (2.8e–07 to 7.2e + 75)	0.099	9.3e + 42 (0.005 to 1.8e + 88)	0.063	1.0e + 43 (0.001 to 9.5e + 88)	0.067
Log leptin						
Intercept	3.89 (1.49 to 10.09)	**0.005**	5.35 (1.84 to 15.57)	**0.002**	3.92 (1.18 to 12.95)	**0.025**
Slope	0.04 (3.6e–46 to 3.4e + 42)	0.948	7.1e–15 (3.0e–62to 1.7e + 32)	0.558	1.1e–10 (7.4e–59 to 1.5e–38)	0.685
Log adiponectin						
Intercept	0.34 (0.12 to 1.00)	0.049	0.53 (0.16 to 1.81)	0.314	0.54 (0.16 to 1.83)	0.321
Slope	45.47 (1.5e–18 to 1.4e + 21)	0.868	76.4 (1.0e–19 to 5.6e + 22)	0.860	0.26 (1.4e–22 to 4.6e + 20)	0.957
Age (years)	—	—	0.94 (0.86 to 1.04)	0.236	0.95 (0.86 to 1.04)	0.266
Education	—	—	1.03 (0.86 to 1.22)	0.765	1.03 (0.87 to 1.22)	0.732
Pre-pregnancy LTPA (no/yes)	—	—	1.76 (0.65 to 4.80)	0.268	1.82 (0.66 to 4.98)	0.246
Parity (n of parturitions)	—	—	1.55 (1.01 to 2.40)	**0.045**	1.52 (0.98 to 2.35)	0.061
Pre-pregnancy energy intake (kcal/day)	—	—	1.00 (1.00 to 1.001)	0.597	1.00 (1.00 to 1.00)	0.662
Glycaemia (mg/dL)	—	—	0.97 (0.92 to 1.02)	0.287	0.97 (0.92 to 1.02)	0.232
Gestational weight gain (kg)	—	—	1.08 (0.97 to 1.21)	0.146	1.09 (0.97 to 1.21)	0.142
Early pregnancy BMI (kg/m^2^)	—	—	—	—	1.07 (0.94 to 1.21)	0.292

^1^LGA was classified according to the international foetal and newborn growth consortium for the 21^st^ Century (Intergrowth-21^st^) curves. ^2^Odds ratio. ^3^p-value refers to the logistic regression.

**Notes:** Intercept variables represent the prediction of the mean exposure level, i.e. biomarkers concentrations when the gestational age was zero and the slope the trend of change in concentrations during pregnancy. **Model 1** included lipids, leptin and adiponectin intercepts and slopes variables; **Model 2** was additionally adjusted for women’s age, education, parity, pre-pregnancy practice of leisure time physical activity, pre-pregnancy energy intake, glycaemia and gestational weight gain. **Model 3** was additionally adjusted for early pregnancy BMI. The models were not adjusted by smoking habit since none of the mothers of LGA smoked during pregnancy.

**Abbreviations:** CI = confidence interval; HDL-c = high-density lipoprotein; LDL-c = low-density lipoprotein; LGA = large-for-gestational-age; LTPA = leisure time physical activity.

LDL-c rate of change per gestational week during pregnancy (slope) was positively associated with BW z-score (β = 0.31; 95%CI: 0.10 to 0.52; p = 0.004), whereas HDL-c rate of change was negatively associated with BW z-score (β = −1.99; 95%CI: −3.30 to −0.68; p = 0.003), even after adjustments. The pregnancy baseline log leptin concentrations were directly associated with BW z-scores after adjustment for confounders (β = 0.43; 95%CI: 0.10 to 0.75; p = 0.010), but the associations were no longer significant after inclusion of early pregnancy BMI in the model (0.19; 95%CI: −0.18 to 0.56; p = 0.306) (Table 5). The log TG and log adiponectin pregnancy baseline concentrations and rates of change during pregnancy were not significantly associated with BW z-score or LGA birth (Tables 4 and 5).

**Table 5:** Linear regression between maternal lipids, log leptin and log adiponectin time trends during pregnancy and birth weight z-score^1^ in a sample of women and their newborns followed at a public health centre in Rio de Janeiro city, Brazil, 2009–2012.

	Birth weight z-score^1^					
	Model 1 (n = 199)		Model 2 (n = 186)		Model 3 (n = 186)	
	β^2^ (95% CI)	p^3^	β^2^ (95% CI)	p^3^	β^2^ (95% CI)	p^3^
HDL-cholesterol						
Intercept	0.06 (0.01 to 0.10)	**0.008**	0.04 (−0.01 to 0.08)	0.095	0.04 (−0.001 to 0.82)	0.054
Slope	−2.15 (−3.47 to −0.83)	**0.002**	−1.85 (−3.18 to −0.53)	**0.006**	−1.99 (−3.30 to −0.68)	**0.003**
LDL-cholesterol						
Intercept	−0.01 (−0.02 to 0.01)	0.376	−0.01 (−0.02 to 0.01)	0.354	−0.01 (−0.02 to 0.01)	0.204
Slope	0.36 (0.15 to 0.58)	**0.001**	0.27 (0.06 to 0.48)	**0.012**	0.31 (0.10 to 0.52)	**0.004**
Log triglycerides						
Intercept	0.11 (−0.46 to 0.68)	0.702	0.21 (−0.38 to 0.80)	0.444	0.11 (−0.48 to 0.68)	0.714
Slope	13.50 (−18.60 to 45.60)	0.408	14.04 (−18.35 to 47.08)	0.387	12.37 (−19.89 to 44.64)	0.450
Log leptin						
Intercept	0.42 (0.10 to 0.74)	**0.010**	0.43 (0.10 to 0.75)	**0.010**	0.19 (−0.18 to 0.56)	0.306
Slope	−15.96 (−53.67 to 21.76)	0.405	−21.97 (−60.43 to 16.49)	0.261	−14.34 (−52.71 to 24.02)	0.461
Log adiponectin						
Intercept	−0.13 (−0.48 to 0.22)	0.469	0.06 (−0.29 to 0.42)	0.736	0.10 (−0.25 to 0.45)	0.555
Slope	11.43 (−5.22 to 28.08)	0.177	10.77 (−5.67 to 27.20)	0.171	8.31 (−8.09 to 24.72)	0.359
Age (years)	—	—	−0.001 (−0.03 to 0.03)	0.963	−0.001 (−0.03 to 0.03)	0.959
Education (years of schooling)	—	—	0.01 (−0.04 to 0.07)	0.707	0.01 (−0.04 to 0.07)	0.699
Smoking habit (no/yes)	—	—	−0.03 (−0.70 to 0.64)	0.924	−0.08 (−0.74 to 0.58)	0.816
Pre-pregnancy LTPA (no/yes)	—	—	0.18 (−0.17 to 0.54)	0.308	0.21 (−0.14 to 0.55)	0.243
Parity (n of parturitions)	—	—	0.14 (−0.004 to 0.30)	0.055	0.13 (−0.02 to 0.29)	0.083
Pre-pregnancy energy intake (kcal/day)	—	—	−0.0001 (−0.003 to 0.0001)	0.246	−0.0001 (−0.0003 to 0.0001)	0.246
Glycaemia (mg/dL)	—	—	−0.01 (−0.02 to 0.01)	0.294	−0.01 (−0.03 to 0.01)	0.185
Gestational weight gain (kg)	—	—	0.04 (0.01 to 0.08)	**0.016**	0.05 (0.01 o 0.09)	**0.009**
Early pregnancy BMI (kg/m^2^)	—	—	—	—	0.06 (0.01 to 0.10)	**0.014**

^1^Birth weight was classified according to the international foetal and newborn growth consortium for the 21^st^ Century (Intergrowth-21^st^) curves. ^2^Linear regression coefficient.^3^p-value refers to the linear regression. **Notes:** Intercept variables represent the prediction of the mean exposure level, i.e. biomarkers concentrations when the gestational age was zero and the slope variables represent the trend of change in concentrations during pregnancy. **Model 1** included lipid, leptin and adiponectin intercepts and slopes variables; **Model 2** was additionally adjusted for women’s age, education, parity, smoking habit, pre-pregnancy practice of leisure time physical activity, pre-pregnancy energy intake, glycaemia and gestational weight gain. **Model 3** was additionally adjusted for early pregnancy body mass index. **Abbreviations:** CI = confidence interval; HDL-c = high-density lipoprotein; LDL-c = low-density lipoprotein; LTPA = leisure time physical activity.

We did not find significant interactions between maternal BMI and lipids, leptin or adiponectin on BW z-score or LGA births in crude or adjusted models (data not shown in tables).

## Discussion

This study has two main findings. First, the HDL-c rate of change during pregnancy was negatively associated with BW z-score and the delivery of LGA newborns, whereas the pregnancy baseline log leptin concentrations, but not the rate of change, were positively associated with these outcomes. Secondly, the LDL-c rate of change over time was positively associated with BW z-score. We did not observe significant associations between gestational changes of log TG and log adiponectin and BW z-score or LGA births in the adjusted models. Moreover, BMI was not an effect modifier of the associations of lipids and leptin with BW z-score and LGA births in our sample.

One limitation of this study was the lack of an oral glucose tolerance test to diagnose gestational diabetes mellitus (GDM), which is strongly associated with BW^24–26^. Thirteen women reported a diagnosis of GDM during pregnancy (6.4%). We compared the analyses with and without these women and found no significant changes in the results. Furthermore, all the adjusted models were controlled for fasting glucose. Multiple regression models were additionally adjusted for other established confounders such as early pregnancy BMI, gestational weight gain (GWG) and smoking habit^3, 5, 7, 27^. A large number of statistical comparisons were carried out, but no adjustments were made for multiple comparisons. This is also a limitation of our study, as it inflates the likelihood of Type I errors. The small number of cases of LGA can be considered another limitation of the study. However, even with a modest sample size, we were able to find statistically significant associations, indicating a stronger relationship between lipids and log leptin and BW z-score/LGA. We only measured total adiponectin in our study. Therefore, the lack of information regarding the high-molecular-weight (HMW) adiponectin, which is the form that has been reported as more strongly correlated to many outcomes such as diabetes and cardiovascular disease^28–30^, limits our conclusions regarding the effect of adiponectin in BW. The measurement of lipid, leptin and adiponectin concentrations during all three trimesters of pregnancy is a strength of this study. Furthermore, BW z-score and LGA were calculated using a population-based international growth curve that has evaluated 20,486 women and their newborns in eight geographically defined urban populations, including Brazil^31^. The study design enabled us to better understand the relationship between the metabolic changes in maternal biomarkers and infant BW. The use of a two-stage procedure^32^ to model the association between time-dependent exposures and a non-time-varying outcome was also a strength of this study. This procedure considers that the repeated measures are correlated, accounts for different rates of change in the exposures and uses their longitudinal predictions to evaluate the association with the outcome.

The HDL-c rate of change during pregnancy was inversely associated with BW z-score and the delivery of LGA newborns. In a case-control study, Kramer *et al*.^33^ also found that HDL-c concentrations were inversely associated with BW, i.e., women who delivered SGA infants had higher concentrations of HDL-c compared to women who delivered AGA infants. Misra *et al*.^14^ evaluated the association between HDL-c concentrations at 10–14, 16–20, 22–26 and 32–36 gestational weeks and BW in 143 American women stratified for pre-pregnancy BMI. The authors found an inverse and statistically significant association at all time points, but only in overweight/obese women. Misra *et al*.^14^ also tested the influence of time-dependent changes in maternal serum HDL-c on BW and concluded that the trajectory of HDL-c change over time was not significantly associated with BW in any BMI category.

In our theoretical model, maternal BMI was considered a possible confounder of the relation between maternal biomarkers (lipids, leptin and adiponectin) and BW since BMI is associated with both exposures and outcome. Previous publications also tested BMI as an effect modifier of the relation between maternal lipids and BW^14^. To test if BMI had the same effect in our sample, we carried out regression models including interaction terms between lipids, leptin and adiponectin (intercept and slope) with early pregnancy BMI. In contrast to Misra *et al*.^14^, we did not find significant interactions in crude or adjusted models between BMI with any of the maternal biomarkers on BW. The difference observed between the two studies can be attributed to BW classifications and the statistical procedure adopted.

We observed that the rate of maternal LDL-c change during pregnancy was positively associated with BW z-score. Although some previous studies did not report this association^6, 14, 17^, Pecks *et al*.^34^ found that the mean LDL-c concentrations were lower in mothers of term (n = 5) and preterm (n = 10) intrauterine growth restricted newborns compared with term (n = 5) and preterm (n = 10) controls, respectively. The association reached statistical significance only between preterm groups. Merzouk *et al*.^35^ found that obese women who gave birth to macrosomic newborns had significantly higher concentrations of LDL-c than those who delivered newborns with a healthy weight.

We performed additional analyses to understand if the results of the present study remained the same when only the AGA subsample was considered. When LGA and SGA cases were simultaneously removed from the analysis, LDL-c and HDL-c lost the significant association with BW z-score. However, when only SGA cases were removed, HDL-c remained significantly associated. When only LGA cases were excluded, only LDL-c remained significantly associated with BW z-score. These analyses revealed that the associations between lipids and BW z-score were partially driven by the extremes of the BW distribution.

Cholesterol is essential for foetal development; it is part of cell membranes, necessary for activation of various signalling pathways and a precursor of steroid hormones. Although most of foetal cholesterol is endogenously obtained by *de novo* synthesis in the liver, there is evidence that maternal cholesterol (exogenous source) crosses the placenta and is important for foetal growth and impacts metabolic function of extraembryonic foetal tissues^10, 36^. Little is known about the biological mechanism by which maternal cholesterol affects BW, but it seems to include altered sterol hormone metabolism and impaired cell cycle and signalling of growth factors (including insulin) and is indirectly by affecting placental transport of nutrients^12, 13, 36^. This mechanism may be involved in the positive association between LDL-c and BW; however, it does not fully explain the inverse association between HDL-c and BW and LGA. We suppose that it may also be related to its antioxidant and anti-inflammatory properties^37^.

We did not find significant associations between maternal serum concentrations of TG and BW or LGA births, in line with results from Retnakaran *et al*.^17^ and Crume *et al*.^38^. TG concentrations are known to affect foetal growth in women who have gestational diabetes^16, 39^; however, in studies with non-diabetic women, it seems not to have the same impact on BW^40^.

We found a positive association between log leptin pregnancy baseline concentrations (intercept) and LGA births and no association between log leptin rate of change during pregnancy and BW z-score or LGA. Experimental studies have indicated a role of leptin in the regulation of the transfer of amino acids and lipids through the placenta^41, 42^; however, the literature remains contradictory, and there is no consensus regarding the association between leptin concentrations and infant BW in humans. Our findings are in line with a study by Shrof *et al*.^23^, which evaluated 1,304 American women and found that those who delivered LGA neonates had higher leptin concentrations than women who delivered term AGA neonates. Franco-Sena *et al*.^43^ evaluated 195 women between 8 and 13 weeks of gestation and found an association between lower concentrations of leptin and a higher risk of SGA. However, other authors have identified an inverse association between leptin concentrations and BW or LGA^17^ or did not find a significant association^44^. One possible explanation for these contradictory results is the difference in sample size or in the times of leptin assessment between these studies.

We did not find significant associations between total adiponectin and BW z-score or LGA births. Ong *et al*.^28^ also did not find significant associations between total adiponectin and BW in a sample of 58 women of Caucasian descent with singleton pregnancies. However, they found a borderline significant association between HMW adiponectin and BW and a significant inverse association between the ratio of HMW to total adiponectin and BW (β = −19.2; p = 0.018). In contrast, Retnakaran *et al*.^17^ found a significant inverse association between total adiponectin in the third trimester and BW in a sample of 422 women without GDM. Although we did not find statistically significant associations between total adiponectin and BW, we observed the same trend reported by Retnakaran *et al*.^17^. We hypothesize that the lack of association observed in our study and in the one conducted by Ong *et al*.^28^ could be attributed to the sample sizes.

This prospective study of low-income women found that maternal HDL-c and LDL-c rates of change during pregnancy were associated with BW z-score, even after adjusting for important confounders such as maternal early pregnancy BMI, GWG and fasting glucose. We also observed that leptin concentrations were positively associated with LGA births. The association between HDL-c and log leptin with LGA births persists even when we enter both variables in the fully adjusted model. There are no established gestational cut-off points for the assessment of lipid concentrations, so any alteration is considered a physiological adaptation of pregnancy. However, our results indicate that lipids and leptin are important to foetal growth and that in the future, the evaluation of lipid changes and leptin concentrations during pregnancy may be used as an additional strategy to screen women at risk of delivering LGA newborns. Although we found relevant associations, additional studies exploring these relationships in different populations and with lager sample sizes are needed to propose specific cut-off points.

## Methods

### Setting and participants

We conducted a prospective cohort study in pregnant women at a municipal health centre in Rio de Janeiro, Brazil from November 2009 to June 2012. Eligibility criteria were: age between 20 to 40 years and pregnancy between 5 and 13 completed weeks of gestation, with no known chronic non-communicable diseases (except obesity).

Women were studied at three time points during pregnancy: weeks 5–13 [median (IQR) = 9.4 (7.8; 11.3)], 20–26 [median (IQR) = 23.4 (22.1; 24.4)], and 30–36 [median (IQR) = 31.8 (30.4; 33.8)] and at a fourth visit between 30 and 45 days post-partum. A total of 322 women were invited to participate and 299 were enrolled in the study. After baseline clinical evaluation 50 women were excluded for the following reasons: a confirmed pre-pregnancy diagnosis of chronic non-communicable diseases, including women with fasting glucose values ≥126 mg/dL at the 1^st^ trimester (n = 12); the presence of infectious or parasitic diseases (n = 9); twin pregnancy (n = 4); and miscarriage (n = 25). We further excluded women with missing values for BW or gestational age at birth (n = 24), with baseline underweight (BMI < 18.5 kg/m²; n = 4) and women with no lipid measurements at the first trimester (n = 22). The baseline sample comprised 199 pregnant women. Thirteen women reported a diagnosis of GDM during pregnancy (6.5%), and two developed hypertension (>140 and/or >90 mmHg systolic and diastolic respectively) during pregnancy.

### Measurements

BW (g) was obtained from the child vaccination booklet at the post-partum interview. We also evaluated BW z-score for gestational age and sex according to the international foetal and newborn growth consortium for the 21^st^ Century (Intergrowth-21^st^) curves^31^. We classified newborns as LGA when the BW, according to the gestational age and sex-specific Intergrowth-21^st^ curves, was above the 90^th^ percentile and as SGA when it was below the 10^th^ percentile.

The gestational age was calculated based on data from the first ultrasonography examination if it was performed prior to 24 weeks of gestation (n = 189; 95.0%). In cases where this measure was unavailable, the date of the last menstrual period was used (n = 10; 5.0%). The gestational age at delivery was calculated based on the date of birth reported at the post-partum visit.

During each trimester of pregnancy, a nurse technician collected two fasting blood samples (2.5 mL) from each woman in vacutainer tubes containing EDTA or separator gel. The samples were centrifuged (5 minutes, 5031 g), and the serum and plasma were immediately stored at −80 °C.

Serum samples were analysed at the Faculty of Pharmacy Clinical Analysis Laboratory (Rio de Janeiro Federal University) for total cholesterol (TC; mg/dL), HDL-c (mg/dL) and TG (mg/dL) by using the enzymatic colorimetric method and an automated analyser (Labmaxplenno®, LabtestDiagnóstica, Minas Gerais, Brazil) and commercial kits (LabtestDiagnóstica). LDL-c (mg/dL) was calculated as follows: TC - HDL-c - (TG/5)^45^.

Plasma leptin (ng/mL) and total adiponectin (µg/mL) concentrations were measured during the three pregnancy trimesters using commercial ELISA kits (Millipore, St. Charles, Missouri, USA), with sensitivities of 0.50 ng/dL and 0.78 µg/mL, respectively.

Maternal characteristics recorded at baseline included age (years), monthly per capita family income (US$), education (years of schooling), current smoking habits (no or yes), alcohol consumption (no or yes), parity (0 or ≥1 parturitions), and pre-pregnancy LTPA (no or yes). The sex of the newborn (male or female) was reported in the post-partum questionnaire.

Maternal body weight (kg) was obtained using a digital scale (Filizzola PL 150, FilizzolaLtda, Brazil). Height was measured twice, using a portable stadiometer at baseline (Seca Ltd., Hamburgo, Germany). Early pregnancy BMI [weight (kg)/height (m)^2^] was calculated based on first trimester weight and height. The cutoff point proposed by the Institute of Medicine^46^ was used to classify the women during early pregnancy as normal weight (18.5 to 24.9 kg/m^2^) or as overweight/obese (≥25.0 kg/m^2^). Anthropometric measures were collected according to standardized procedures and performed by trained interviewers^47^.

GWG (kg) was calculated as the difference between the last weight measured before delivery (mean gestational age = 37.9 weeks; SD = 2.3) and the first trimester weight (mean gestational age = 9.6 weeks; SD = 2.2).

Fasting glucose (mg/dL) was measured in all pregnancy trimesters using the glucose oxidase-peroxidase enzymatic colorimetric method and a Wiener Lab kit (Rosario, Argentina).

Total energy intake was assessed using a semi-quantitative food frequency questionnaire (FFQ) validated for the adult population of Rio de Janeiro^48^. The FFQ was composed of 81 food items, eight frequency options and household measure portion options. The FFQ was administered at the first gestational trimester (5–13 weeks of gestation) and referred to food intake 6 months prior to pregnancy. For statistical analysis, frequency options were transformed into daily frequencies and household measures into grams (g) or milliliters (ml)^49^. The daily amount consumed (g or ml/day) of each food item was obtained by multiplying the daily frequency (3x/day; 2 to 3x/day; 1x/day; 5 to 6x/week; 2 to 4x/week; 1x/week; 1 a 3x/month and never or almost never) per portion size. The Brazilian Food Composition Table (TACO)^50^ was used as the main database to determine food nutritional composition and The National Nutrient Database for Standard Reference provided by the United States Department of Agriculture^51^ was considered as a secondary option when a food item was not available in the TACO database.

### Ethics

The research ethics committee of the Municipal Secretary of Health of Rio de Janeiro Municipality (Protocol number: 0139.0.314.000-09, approved on 13 August 2009) approved the study protocol. All participants signed a consent agreement, which was obtained freely and spontaneously, after all necessary clarifications had been provided. All ethical procedures of this study involving human beings followed the Brazilian Resolution 196/96.

### Statistical analysis

General characteristics of the sample were described as the means and standard deviations (SD) for continuous variables and proportions (%) for categorical variables. Student’s unpaired t test was used to compare means, and the chi-square test was used for proportions. The mean variation (SD) between the first and the third trimester biomarkers values was calculated.

To evaluate the association between biomarker changes during pregnancy and BW z-scores and LGA, we used a two-stage method. (1) We constructed a linear mixed-effect model (LME) for each exposure (maternal lipids, leptin and adiponectin) including gestational age at sampling as fixed and random effects and estimated the best linear unbiased prediction (BLUP) of random coefficients. The predicted intercept refers to the mean lipid, leptin or adiponectin exposure level, i.e., the biomarker concentrations when the gestational age was zero, and was labelled as the pregnancy baseline concentration. The predicted slope refers to the rate in concentration changes per gestational age during pregnancy. (2) The BLUP predicted intercept and slope were simultaneously included as continuous predictors in linear and logistic regression models having BW z-score and LGA as outcomes, respectively. This approach considers that repeated measures are correlated and estimates time trends of exposure even for women with missing values across pregnancy, increasing the power of the analysis^32^. Since the LME model assumes that the dependent variable is normally distributed, we have log transformed variables with skewed distribution (TG, leptin and adiponectin).

The modelling process was performed in three steps. We constructed three linear (outcome: BW z-score) and three logistic (outcome: LGA) regression models to test the association between maternal biomarkers (lipids, leptin and adiponectin) and BW z-score or LGA births, reporting the regression coefficient (β) and odds ratio (OR), respectively, and their 95% confidence intervals (95% CI). In the first models, lipids, leptin and adiponectin intercept and slope variables were included together in the same model. The second models were additionally adjusted for age (years), education (years of schooling), pre-pregnancy LTPA (no/yes), smoking habit (no/yes), parity (number of parturitions), total pre-pregnancy energy intake (kcal/day), fasting glucose (mg/dL) and GWG (kg). In the third models, the early pregnancy BMI variable was added. We also tested if there was an interaction between maternal BMI and lipids, leptin or adiponectin on BW z-score and LGA births. The adjustment variables were chosen based on the biological plausibility of the association.

Since the models were adjusted for variables that could be correlated with each other, we tested the correlation between them. Variables with strong correlations (Pearson or Spearman coefficient ≥0.7) were candidates to be excluded. The TC was not included in the fully adjusted models due to its strong correlation with HDL-c and LDL-c.

We further investigated the occurrence of multicollinearity in the full models using the variance inflation factor. We predicted residuals and fitted values of the outcomes for our final models. We checked normality of the residuals and constructed two-way scatter plots between the residuals and predicted values of BW to detect outlying observations and to check the assumption of constant variability of outcomes across values of exposure (homoscedasticity) and scatter patterns of the residuals^52^.

Effect plots were created to present the longitudinal prediction of maternal biomarker changes during pregnancy for the total sample and according to BW categories (SGA or AGA vs. LGA). In these plots, the longitudinal prediction and 95% CI (black line and shaded area, respectively) represent the effect of gestational age and quadratic gestational age, when applicable, on maternal lipids, leptin and adiponectin. The effect plots were adjusted for age, education, smoking habit, pre-pregnancy LTPA, pre-pregnancy energy intake, glycaemia, parity, early pregnancy BMI, and gestational weight gain, but no adjustments were made for multiple comparisons.

Statistical analyses were performed using Stata Data Analysis and Statistical Software (STATA) version 12.0 (Stata Corp., College Station, Texas, USA). Values were considered statistically significant when the p-value was lower than 0.05.
